# The chloroplast genome of the moss *Haplocladium microphyllum*, first in family Thuidiaceae

**DOI:** 10.1080/23802359.2020.1789006

**Published:** 2020-07-15

**Authors:** Lihui Mao, Huaqiao Ding, Qing Dong, Danqing Tian

**Affiliations:** Zhejiang Institute of Landscape Plants and Flowers, Hangzhou, China

**Keywords:** Bryophytes, Thuidiaceae, chloroplast, next-generation sequencing

## Abstract

Bryophytes are a highly diverse group containing more than 12,800 species. *Haplocladium microphyllum* is in a large moss belonging to the family Thuidiaceae. We report the complete chloroplast (124,478 bp) genome sequence of *H. microphyllum*, it includes a pair of inverted repeat regions (IRs, 9727 bp), one large single-copy (LSC, 86,528 bp) region, and one small single-copy (SSC, 18,496 bp) region. Besides, the complete chloroplast genome contains 134 genes in total, including 88 protein-coding genes, 38 tRNA genes, and eight rRNA genes. Phylogenetic analysis showed that *H. microphyllum* has the closest relationship with *Sanionia uncinata* in Amblystegiaceae. Our study lays a foundation for further research like speciation of this species and the phylogeny of the Thuidiaceae family.

Plants of *Haplocladium microphyllum* were collected in Wangcun Village, Linpu Town, Xiaoshan District, Hangzhou, China (30.069°N, 120.232°E) in December 2019 (specimen deposited in SZG, number: Mao201912011). The genomic DNA was extracted with plant genomic DNA kit (Tiangen Biotech, Beijing, China) and sequenced using the Illumina NovaSeq platform following the manufacturer’s recommendations. The chloroplast genome was assembled with SPAdesv3.10.1 (Bankevich et al. 2012) and annotated with blast v2.2.2 (https://blast.ncbi.nlm.nih.gov/Blast.cgi) and hmmer v3.1b2 (http://www.hmmer.org/). The annotated genomic sequence has been submitted to NCBI GenBank (MT385397).

In order to confirm the phylogenetic position of *H. microphyllum*, a maximum-likelihood analysis was performed by RAxML v8.2.10 (Stamatakis 2014) using GTR model with 1000 bootstrap replicates. Species used in phylogenetic analysis are 11 bryophyte species and one fern as outgroup.

The complete chloroplast genome is 124,478 bp in length and has 127 genes in total, including 82 protein-coding genes, 37 tRNA genes, and 8 rRNA genes. Phylogenetic analysis showed that *H. microphyllum* has the closest relationship with *S. uncinata* in Amblystegiaceae, *O. rogeri* and *N. obtusifolia* in the same family Orthotrichaceae with the closet relationship, *T. lepidozioides* has the farthest relationship with other mosses (Figure 1). Those above indicate phylogenetic analysis of mosses based on chloroplast genome is credible.

**Figure 1.**
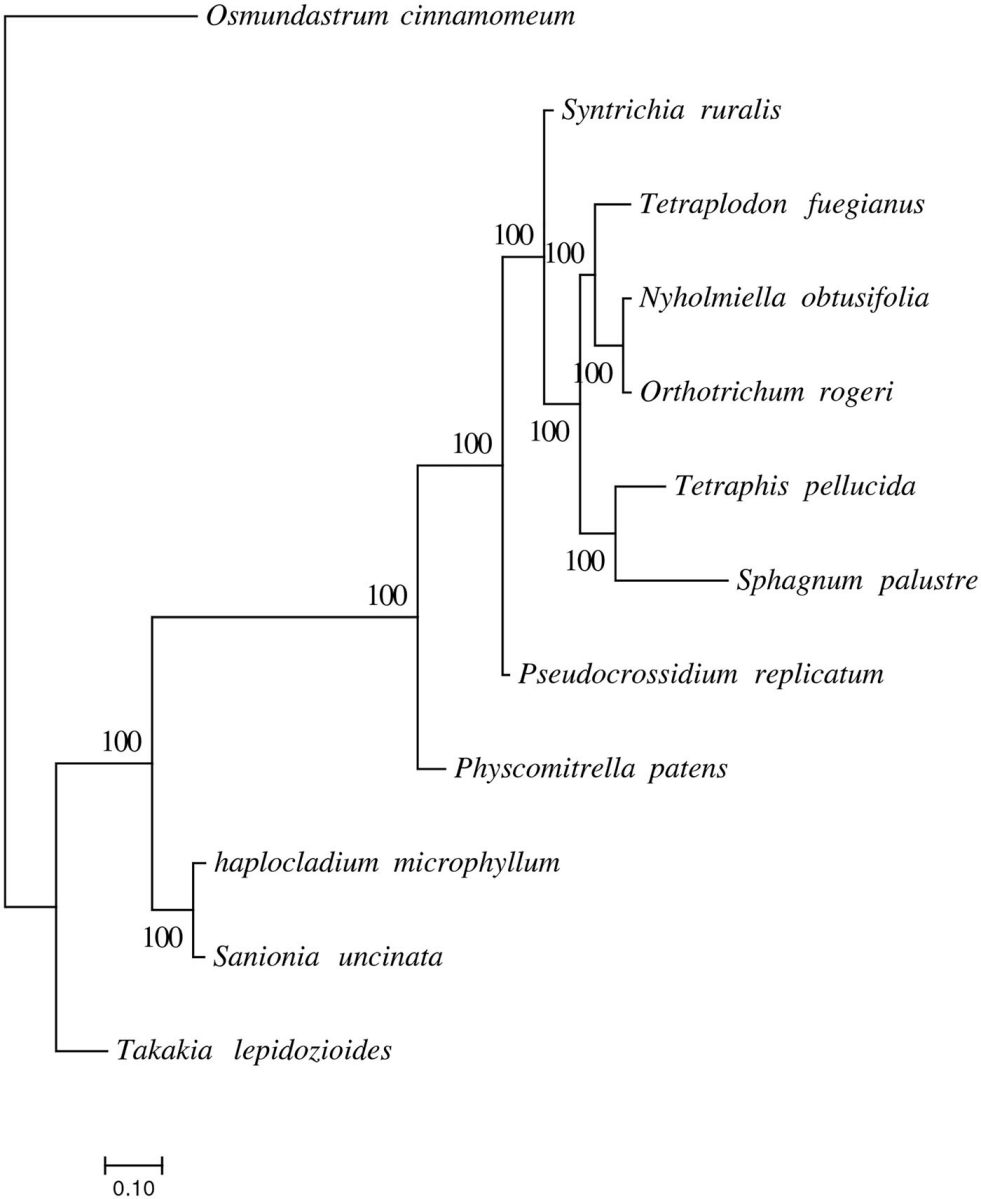
Molecular phylogenetic analyses of *H. microphyllum* and other mosses. *Tetraplodon fuegianus* (KU171381.1), *Tetraphis pellucida* (NC_024291.1), *Takakia lepidozioides* (NC_028738.1), *Syntrichia ruralis* (NC_012052.1), *Sphagnum palustre* (NC_030198.1), *Sanionia uncinata* (NC_025668.1), *Pseudocrossidium replicatum* (MG132071.1), *Physcomitrella patens* (NC_005087.2), *Orthotrichum rogeri* (NC_026212.1), *Nyholmiella obtusifolia* (NC_026979.1), and *Osmundastrum cinnamomeum* (NC_024157).

*Haplocladium microphyllum* is an intercontinental distribute species, it has many closely related species like *H. discolor*, *H. perparaphyllum*, *H. angustifolium*, and *H. strictulum*, also many varieties for this species such as *H. microphyllum*, var. *capillata*, *H. microphyllum* var. *latifolium*, *H. microphyllum* var. *cryptocoleum*. The differences between *H. virginianum* and *H. microphyllum* are smaller plants, rigid branches, more dense branch leaves for the former, whether it belongs to a single species or a variety in morphological classification is controversial (Gier 1980). *Haplocladium microphyllum* and its related species represent nearly half of the world *Haplocladium* species (16 recognized), it is not only important but also difficult for the taxon of this genus to clarify the classification of the *H. microphyllum* complex and related species.

Compared with morphological studies, molecular sequences allow us to recognize the diversity bryophytes more quickly and accurately, especially some widely distributed species or complexes (Hutsemékers et al. 2012; Lang et al. 2015). SSR molecular markers are widely used in species identification and phylogenetic analysis for their high polymorphism and stable amplification. In this work, 408 SSRs are found in the cp genome, among them 99 tri-nucleotide, tetra-nucleotide, and penta-nucleotides repeats are 99, 17, and 5, respectively. cpDNA sequences like trnL-F, rps4, trnG, and psbA-trnH are already successfully applied in related moss species classification (Lang et al. 2015; Vigalondo et al. 2019). Many work on angiosperm confirmed other cpDNA genes also can be candidates and then test the polymorphism finally used to delimitation and phylogenetic study of closely affinity species (Nguyen et al. 2017; Kim et al. 2018).

The results of this work can provide a basis for speciation and classification for *H. microphyllum*. The complete chloroplast genome data can also lay the foundation for the phylogenetic and taxonomic studies of Thuidiaceae which is one of the most species rich family in mosses.

## Data Availability

The data that support the findings of this study are openly available in GenBank of NCBI at https://www.ncbi.nlm.nih.gov, reference number MT385397.
